# Teclistamab‐Induced Remission of Chronic Immune Thrombocytopenia in a Patient With Multiple Myeloma

**DOI:** 10.1002/jha2.70062

**Published:** 2025-05-25

**Authors:** Renata Quevedo‐Salazar, José Miguel Yáñez‐Reyes, Elías Eugenio González‐López, Ana Varela‐Constantino, Andrés Gómez‐De León, Xitlaly Judith González‐Leal, David Gómez‐Almaguer

**Affiliations:** ^1^ Tecnologico de Monterrey Internal Medicine, Monterrey Nuevo Leon Mexico; ^2^ Clinica Gomez Almaguer, Hematology Monterrey Nuevo Leon Mexico; ^3^ Hospital Universitario Dr. Jose Eleuterio Gonzalez Universidad Autonoma de Nuevo Leon, Hematology Monterrey Nuevo Leon Mexico

## Abstract

We report the case of a 76‐year‐old woman with refractory immune thrombocytopenia (ITP) and multiple myeloma (MM) who achieved remission of both conditions following teclistamab treatment. After failing multiple ITP therapies, her platelet count continued to decline. Initiation of teclistamab led to a rapid and sustained platelet increase, alongside MM response with measurable residual disease (MRD) negativity. Notably, remission was achieved after only four full doses, and the patient has remained in complete remission since. This case highlights teclistamab as a potential treatment for refractory ITP, suggesting a novel role for B‐cell maturation antigen (BCMA)‐targeted therapies in autoimmune diseases.

**Trial Registration:**

The authors have confirmed clinical trial registration is not needed for this submission.

## 1 Introduction

Primary immune thrombocytopenia (ITP) is an autoimmune disorder characterized by platelet destruction and impaired megakaryocyte production. A chronic course occurs in up to two‐thirds of adults [1], with approximately 10% developing refractory ITP [2] unresponsive to standard therapies such as corticosteroids, intravenous immunoglobulin (IVIG), rituximab, and thrombopoietin receptor agonists [3]. Combination strategies targeting multiple pathways show promise, though their efficacy remains variable. Novel agents like fostamatinib and avatrombopag have expanded treatment options [4, 5], while daratumumab has shown potential in refractory cases [6].

Teclistamab, a bispecific antibody targeting B‐cell maturation antigen (BCMA) on plasma cells and CD3 on *T* cells, has demonstrated efficacy in relapsed/refractory multiple myeloma (MM) [7]. Its mechanism suggests a potential role in ITP, mitigating platelet destruction by depleting plasma cells hypothesized to produce antiplatelet autoantibodies. Recent evidence supports its broader application in autoimmune diseases, including systemic lupus erythematosus [8] and TEMPI syndrome [9].

## 2 Case Presentation

A 67‐year‐old woman presented in 2016 following a 1‐year history of Raynaud's phenomenon, progressive ecchymosis, and hyperpigmented lesions. Thrombocytopenia secondary to vasculitis was initially suspected, prompting a comprehensive workup. Initial laboratories showed a haemoglobin (Hb) level of 13.9 g/L, white blood cells (WBC) of 2.9 × 10^9^/L, and a platelet count of 7 × 10^9^/L. The basic metabolic panel was unremarkable. Severe thrombocytopenia prompted hospitalization and treatment with high‐dose dexamethasone (40 mg/day for 4 days) and low‐dose rituximab (100 mg weekly for four doses); achieving an initial response (40 × 10^9^/L). In March 2016, a bone marrow biopsy ruled out alternative hematologic disorders, confirming primary ITP as per the American Society of Hematology 2011 guidelines [10].

Despite treatment, she experienced recurrent exacerbations, requiring multiple therapies without sustained remission. In 2019, she developed severe, progressive lumbar pain. An MRI revealed fractures of the T9, L1, L2, L4, and L5 vertebral bodies. Further workup revealed a monoclonal IgG kappa band (2.33 g/dL) on serum protein electrophoresis and kappa light chain proteinuria. A bone marrow aspirate and biopsy showed > 10% plasma cells, leading to a diagnosis of MM. Treatment with bortezomib, lenalidomide, and dexamethasone (VRd) began in May 2019, achieving a partial response, with platelet counts stabilizing between 94–150 × 10⁹/L. In December 2019, due to bortezomib‐induced neuropathy, therapy transitioned to daratumumab‐Rd, leading to a very good partial MM response, with monthly daratumumab maintenance. She was ineligible for autologous stem cell transplantation.

In 2023, she experienced a relapse of ITP (platelet count of 8 × 10⁹/L), initially responding to corticosteroids and eltrombopag, achieving remission by May 2023 (196 × 10⁹/L). This allowed gradual tapering of both steroids and eltrombopag. However, by June 2023, she developed petechiae and ecchymosis, with a platelet count of 17 × 10⁹/L, requiring an increased eltrombopag dose; subsequent dose reductions resulted in platelet counts as low as 10 × 10⁹/L. In April 2024, she experienced biochemical MM progression while on daratumumab maintenance. Having progressed on prior lines of therapy and with no access to chimeric antigen receptor (CAR) *T*‐cell therapy in Mexico, she was started on teclistamab in June 2024. The drug was administered subcutaneously using a step‐up dosing regimen (0.03 mg/kg followed by 0.6 mg/kg), then transitioned to a full dose of 1.5 mg/kg weekly for four doses, with the last given in July 2024. After one cycle, platelet count rose from 51 × 10⁹/L to 256 × 10⁹/L, achieving complete ITP remission. In addition, she attained a stringent complete response in MM with undetectable measurable residual disease by next generation flow cytometry by September 2024.

Given her substantial clinical improvement and awareness of potential therapy‐related infection risk, she opted to discontinue teclistamab. At 6 months' follow‐up, she remained clinically stable and in complete remission for both conditions (January 2025). By then, her complete blood count (CBC) showed a Hb of 13.8 g/L, WBC of 6.25 × 10^9^/L, and a platelet count of 248 × 10^9^/L. Free kappa light chain levels were 0.117 mg/dL, while free lambda light chain levels were below 0.131 mg/dL, with no M spike on serum protein electrophoresis. Her most recent CBC (April 2025) showed a Hb of 14 g/L, WBC of 6.67 × 10^9^/L, and a platelet count of 231 × 10^9^/L.

## 3 Discussion

**FIGURE 1:**
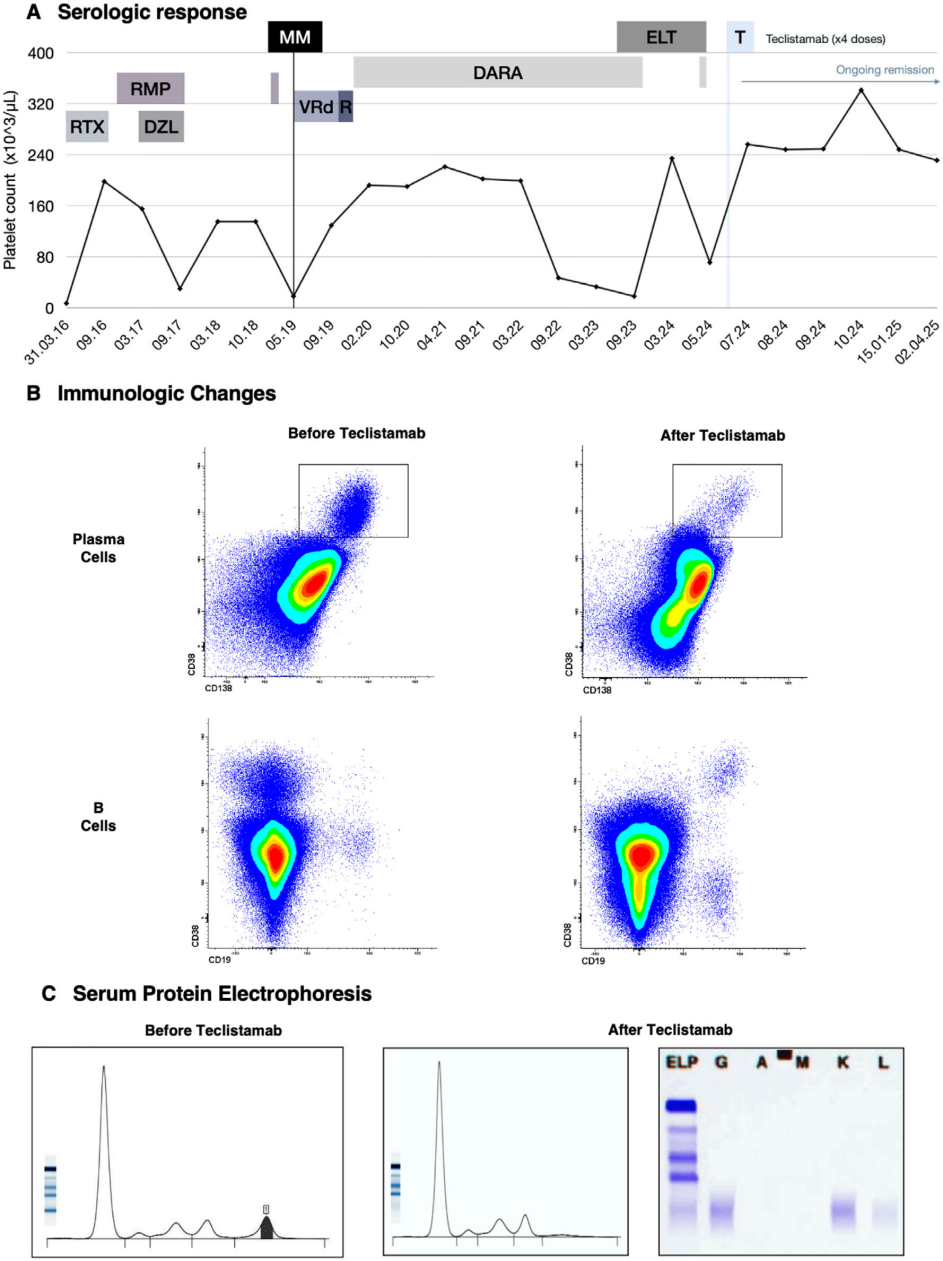
Responses to BCMA‐directed bispecific antibody therapy in a patient with chronic immune thrombocytopenia and concomitant multiple myeloma. Panel A Serologic response. Patient's clinical course with platelet count (x10^3^/µL) on the *y*‐axis and time (dates) on the *x*‐axis. Different treatments are labeled along the timeline (DARA: daratumumab, DZL: danazol, ELT: eltrombopag, MM: multiple myeloma, R: lenalidomide, RMP: romiplostim, RTX: rituximab, T: teclistamab, VRd: bortezomib, lenalidomide, dexamethasone). Panel B immunologic changes. Flow cytometry: Before teclistamab: 6% of monotypic plasma cells are observed with Kappa light chain restriction in the cytoplasm, consistent with clonal plasma cell involvement in multiple myeloma. After teclistamab: only 0.02% of polytypic plasma cells with a normal phenotype are observed. This finding is compatible with no measurable residual disease (MRD). Panel C Serum protein electrophoresis: Before teclistamab: a monoclonal spike (M‐spike) is visible. After teclistamab: an IgG Kappa restriction band without a detectable M‐spike.

The coexistence of ITP and MM in our patient underscores the complex interplay of immune dysregulation in plasma cell disorders. Without serum protein electrophoresis at the time of ITP diagnosis, the presence of an underlying monoclonal gammopathy of undetermined significance (MGUS) cannot be entirely ruled out in retrospect. However, the absence of plasma cell infiltration in the initial bone marrow biopsy makes this scenario unlikely. The rapid and sustained remission of both ITP and MM after only four full doses of teclistamab—a notably short course—is particularly noteworthy (Figure 1).

While ongoing trials evaluate anti‐BCMA CAR‐T cell therapy for relapsed/refractory ITP [11, 12], teclistamab's role in ITP has yet to be explored in clinical trials, to the best of our knowledge. Given her prior progression on daratumumab, teclistamab's more profound depletion of mature *B* cells and normal plasma cells—compared to anti‐GPRC5D bispecific antibodies [13]—likely contributed to the response. Even though Teclistamab‐induced hypogammaglobulinemia required IVIG (400 mg/kg) to mitigate infection risk, the platelet increase preceded IVIG administration and occurred at a dose significantly lower than that typically used for refractory ITP (1–2 g/kg) [14]; reinforcing teclistamab's role as the primary driver of response.

## 4 Conclusion

This report adds to the growing real‐world evidence supporting teclistamab's role in autoimmune disease. Future studies should determine whether teclistamab's effects are solely due to plasma cell depletion or if additional immunomodulatory mechanisms are involved. A deeper understanding of its long‐term efficacy and safety in autoimmune cytopenias could further refine its therapeutic application.

## Author Contributions

All authors contributed to manuscript drafting, revision, and approval of the final version.

## Ethics Statement

Ethics approval was obtained in accordance with institutional guidelines.

## Consent

Written informed consent was obtained from the patient for publication.

## Conflicts of Interest

The authors declare no conflicts of interest.

## Data Availability

All relevant data supporting the findings are available upon request.
